# Perceptions of Health in the Denver Refugee Community: A Mixed-Methods Study

**DOI:** 10.3390/ijerph22121876

**Published:** 2025-12-17

**Authors:** Katherine Boyd, Jini Puma, Anne Lambert-Kerzner, Benjamin C. Ingman, Maytham Alshadood, Carol E. Kaufman

**Affiliations:** 1 Department of Community and Behavioral Health, Colorado School of Public Health, University of Colorado Anschutz, 13001 East 17th Avenue, B119, Aurora, CO 80045, USA; 2 Rocky Mountain Prevention Research Center, Colorado School of Public Health, University of Colorado Anschutz, 13001 East 17th Avenue, B119, Aurora, CO 80045, USA; 3 Surgical Outcomes and Applied Research (SOAR) Collaborative, School of Medicine, University of Colorado Anschutz, 13001 East 17th Place, Campus Box C290, Aurora, CO 80045, USA; 4 Institute on the Environment (IonE), University of Minnesota, 954 Buford Ave, Ste 425, Saint Paul, MN 55108, USA; 5 Independent Researcher, Denver, CO 80202, USA; 6 Centers for American Indian and Alaska Native Health, Colorado School of Public Health, University of Colorado Anschutz, 13055 East 17th Avenue, F800, Aurora, CO 80045, USA

**Keywords:** refugee health, social determinants of health, mixed methods, community-engaged research

## Abstract

Refugees often face significant barriers to healthcare access and integration, contributing to poor health outcomes. Although perceptions of health are known predictors of self-reported health status, little is known about how refugees themselves conceptualize health. This study employed a community-engaged, transformative mixed-methods design to explore refugee health perceptions in the Denver-metro area. Data collection included 149 surveys and 27 interviews with refugees and asylum seekers conducted between November 2018 and March 2019. Hierarchical linear regression was used to assess associations between social determinants of health (SDoH) and self-reported health, while qualitative data were analyzed using a constant comparative approach. The final regression model explained 75.8% of the variance in self-reported health (R^2^ = 0.758, *p* < 0.001). Significant predictors included country of origin (Burma: −3.419, *p* = 0.030; Somalia: −9.155, *p* < 0.001), age (1.901, *p* < 0.001), sex (male: −3.252, *p* < 0.001), and education level (−0.999, *p* < 0.001). Qualitative findings revealed themes such as health as the ability to live life and health as happiness, each shaped by cultural context, community connectedness, and perceptions of safety. Integrating these findings highlights how structural conditions and culturally rooted understandings of well-being intersect to shape refugee health after resettlement. This study underscores the need for public health and clinical interventions that center refugee-defined priorities and suggests future research should incorporate constructs, such as happiness and culturally grounded notions of safety, that emerged as central to health in this study.

## 1. Introduction

The world is currently witnessing an unprecedented refugee crisis, with more than 100 million refugees and asylum seekers forcibly displaced globally [1,2]. Resettled refugees face disproportionately poorer health outcomes including increased morbidity, poorer health habits, and decreased life expectancy [3,4,5]. Structural barriers such as limited access to linguistically and culturally appropriate care, unstable employment, discrimination, and restrictive immigration policies further entrench these inequities. [3,4,6,7]. In early 2025, the abrupt suspension of the U.S. Refugee Admissions Program halted the resettlement of over 10,000 refugees. This policy shift has intensified uncertainty, legal precarity, and health risks among those awaiting resettlement, highlighting the importance of refugee-informed evidence to inform responsive policies, guide service delivery, and mitigate the long-term consequences of displacement and delayed integration [8]. Understanding how refugees themselves define and experience health is critical for designing policies and services that respond to these intersecting vulnerabilities.

The social factors that determine health, or social determinants of health (SDoH), are shaped by both broader social and policy factors (such as access to safe environments and employment), as well as individual factors (such as age and sex) [9]. For resettled refugees, many of these factors impact both health outcomes and integration [10]. In addition, diverse sociocultural and historical backgrounds lead different refugee communities to hold divergent beliefs about health, illness, and care-seeking [11]. Understanding these factors from the perspective of refugees, as they relate to self-reported health status after resettlement, can provide deeper insight into their health experiences and priorities [12]. Refugee perceptions of health are shaped by both universal factors and community- and culture-specific experiences. For example, refugees from Burma, Somalia, and Iraq represent highly diverse sociocultural, linguistic, and historical contexts, each with distinct traditions, social networks, and health beliefs [11,13]. These variations illustrate the importance of understanding how refugees interpret and communicate about health within their own cultural frameworks. This study conceptualizes health in broad, holistic terms—viewing health as not merely the absence of disease but a state of overall physical, mental, and social well-being integrated with daily life [14,15]. However, few comparative studies have examined such broad perceptions of health across different refugee groups. This gap in knowledge poses a challenge for developing effective, culturally responsive health programs.

This study utilized a concurrent mixed-methods research (MMR) approach [16,17] to explore perceptions of refugee health in the Denver refugee community. MMR has been shown to be particularly applicable for migration research, where complex social dynamics and structural power differentials call for flexible, transdisciplinary and new approaches to conceptualizing, theorizing, and conducting research [18]. Transformative MMR designs aim to improve health outcomes by generating solutions in partnership with research participants who are positioned to engender ripple effects in the broader refugee community [19]. Additionally, community-engaged research has been identified as an approach to address health disparities [20,21,22], which are well-documented in the refugee community [3,4,5]. Members of the refugee community were engaged as partners throughout the research process to ensure cultural relevance and build trust—from refining research questions and data collection to interpretation of results.

**Figure 1:**
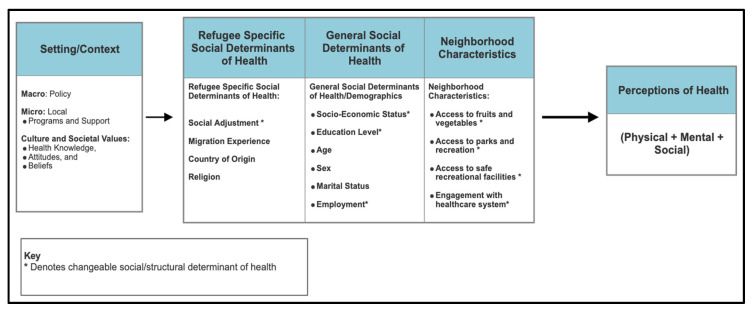
Perceptions of health in the refugee and asylum-seeking community: A social ecological exploratory framework.

Social ecological perspectives help to emphasize the interactions and contexts of behavior while considering different levels of influence, starting with understanding interpersonal health behavior and moving outward to study community-level factors [23]. Social ecological frameworks consider the complex interplay between individual, relationship, community, and societal factors [24]. When applied to interventions and programs, social ecological frameworks center the community as a unit of identity, consider decentralized treatment and intervention services, and, in the context of refugee health, can promote the sustainability of culture while integrating into the resettlement country [25]. This study attended to the ecological perspective by examining SDoH specific to the refugee community, then incorporating general SDoH into the model and finally including neighborhood characteristics into the model (Figure 1). By situating refugee health perceptions within this broader context, the study aimed to illuminate how structural conditions and cultural contexts intersect in shaping health for these communities.

Main Aim and Significance: Despite extensive literature documenting refugee health disparities and social determinants of health, relatively little research centers on refugees’ own perceptions of health or examines how these subjective understandings align with quantitative measures of self-reported health. This gap is particularly evident in studies comparing multiple refugee communities within a single resettlement context. Addressing this gap, the present study explores how refugees from Burma, Somalia, and Iraq living in Denver define health and identify the factors they believe most shape their well-being after resettlement. Insights from this study can inform the design of more culturally responsive public health interventions and policies that reflect the lived experiences of diverse refugee populations.

## 2. Materials and Methods

### 2.1. Study Design and Setting

**Figure 2:**
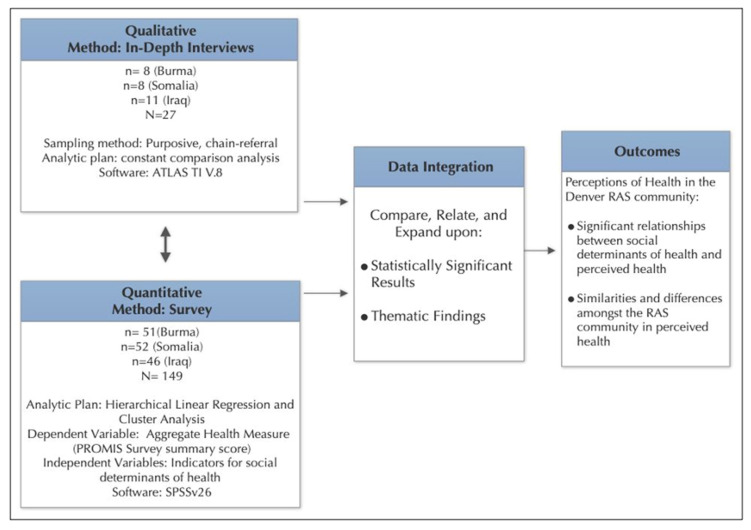
Concurrent mixed method design to explore perceptions of health in the Denver refugee community.

This study employed a concurrent mixed-methods research design [16,17] to explore perceptions of health among resettled refugee communities in the Denver metro area (Figure 2). Quantitative and qualitative data were collected simultaneously to allow for triangulation and a richer understanding of both contextual factors and subjective descriptions of health. Data collection was conducted between November 2018 and March 2019, and all procedures were approved by the Colorado Multiple Institutional Review Board (COMIRB #18-1399). This study was completed prior to the availability of generative artificial intelligence tools and did not use GenAI for any aspect of the study design, analysis, or interpretation.

The study recruited refugees aged 18 years and older from three countries of origin—Burma (Myanmar), Iraq, and Somalia—who had resettled in the Denver metro area from 2008–2018. The state of Colorado resettles approximately 2% of refugee arrivals nationally [13], and approximately 90% of those arrivals settle in the Denver metro area. These three groups were selected based on their high representation in local refugee resettlement (49% between 2008–2018) [26] and the diverse experiences of displacement affecting each group [2,27]. These groups had sufficient population size and social-network density to support sampling, which relied on peer recruitment chains. Smaller and more recently resettled groups were not included because study resources limited the number of languages in which survey and interview materials could be translated. As a result, findings may not fully represent the experiences of smaller or emerging refugee communities in the region.

### 2.2. Community-Based Research Network (CBRN)

A community-based research network (CBRN) was formed in the Denver metro area to engage refugees, service providers, resettlement agencies, and others identified by the community to increase capacity to engage in community-engaged research. The CBRN was comprised of twenty-one members, including refugees, healthcare providers, refugee service providers, public officials, and university researchers and staff. Members represented a range of local partners, including voluntary resettlement agencies, refugee screening clinics, community-based and non-governmental organizations, foundations, and the university. The network met monthly between April 2018 and June 2019 with the goals of collaboratively identifying and prioritizing health research topics and supporting culturally appropriate dissemination and engagement strategies. CBRN members piloted the survey and interview tools; reviewed and revised translations of the consent forms, surveys, and interview guides; advised on recruitment strategies; and referred participants through their community networks. Several trained CBRN members also served as interpreters for qualitative interviews. Study documents were modified throughout data collection based on CBRN recommendations. For example, adjusting professional translations to align with the reading levels and preferences of the community; when differences in perspective arose between the research team and CBRN members (i.e., or regarding culturally appropriate terminology or how to frame sensitive questions), they were addressed through collaborative dialogue and resolved by reaching group consensus.

### 2.3. Quantitative Methods

#### 2.3.1. Recruitment and Survey Administration

Survey recruitment followed a modified respondent-driven sampling (RDS) strategy [28,29] using community-nominated “seeds” from each refugee group to initiate peer recruitment. In this study, “seeds” were trusted community members from each participating country of origin who served as the initial points of contact for sampling. These individuals were members of the CBRN or had strong social ties within their communities, enabling them to comfortably and credibly refer peers into the project. Seeds distributed coupons with the researcher′s contact information to eligible peers. Surveys were administered in English, Somali, Burmese, Karen, or Arabic, with informed consent obtained in the participant’s preferred language. Verbal informed consent was obtained from all participants prior to data collection. The quantitative component of the study was approved by the Colorado Multiple Institutional Review Board (COMIRB #18-1399). All survey and consent materials were professionally translated into Somali, Burmese, Karen, and Arabic. Translations were piloted with members of the Community-Based Research Network (CBRN), who reviewed each version for accuracy, cultural relevance, and clarity. Several professional translations required modification because the initial wording was overly formal; these were revised collaboratively with CBRN members to ensure accessibility and cultural appropriateness. Participants were informed about the purpose of the study, their rights as participants, and the voluntary nature of their participation before any data were collected. Surveys were administered in person by the research team in locations preferred by participants, including homes, community centers, and community events. All surveys were completed on paper. Participants were compensated $10. Surveys took approximately 20 min to complete and were administered on paper. Following data collection, the researcher manually entered all paper responses into Qualtrics to create the analytic dataset [30]. Sample size was determined using Green’s rule of thumb (5–20 cases per independent variable) [31], targeting 150 participants based on 15 independent variables. The final sample size was 149.

#### 2.3.2. Dependent Variable

Health status was measured using the PROMIS Global Health survey [32], which assesses perceived physical, mental, and social well-being. PROMIS has been validated as a proxy for psychological and physiological functioning [33,34]. Participants rated statements such as “In general, how would you rate your mental health?” on a 5-point Likert scale. Exploratory factor analysis confirmed construct validity [35] and the internal consistency of the PROMIS scale was high (Cronbach’s α = 0.918) after removing the fatigue item to improve reliability. To preserve partial data, we used the SUM function, which calculates the total score based on available (non-missing) item responses rather than excluding participants with any missing values. As a result, the observed range in the analytic sample ranged from 7 (low self-reported health status) to 39 (high self-reported health status), which differs from the theoretical range (9 to 45), reflecting variation in the number of items completed by each respondent. The resulting sum score of the 9-item scale was used as the dependent variable for all subsequent quantitative analysis.

#### 2.3.3. Independent Variables

Fifteen independent variables were grouped into three domains: refugee-specific SDoH, general SDoH, and neighborhood characteristics. Figure 1 graphically depicts the indicators included in the model, starting with what is closest to the refugee experience (e.g., social adjustment and migration experience), subsequently accounting for the relationship between general social determinants of health (e.g., age, sex and marital status) and ending with environmental characteristics (e.g., access to parks and recreation and healthcare) that may be associated with self-reported health status.

Refugee-specific SDoH was assessed by asking about social adjustment, migration experience, country of origin and religion. All variables were coded as categorical. Social adjustment: was measured by two questions: “Thinking about your time before you resettled, would you say most people you know would categorize a household like yours as having lower income, lower-middle, middle, upper-middle, or upper income?” And “In your current home now in Denver, would you say most people you know would categorize a household like yours as having lower income, lower-middle, middle, upper-middle, or upper income?” [36] The difference in responses were then calculated and recoded as follows negative (or downward) change in social adjustment (0); no change (1); and a positive change in social adjustment (2). Migration experience was assessed by asking the length of time in a refugee camp [3]: “How many years did you spend in a refugee camp” no years in a refugee camp (0); 1–5 years (1); 6–10 years (2); 11–15 years (3); and 16+ years in a refugee camp (4). We also asked length of time in the U.S. to assess migration experience: “How long have you been in the U.S.?” less than one year (1), 1–3 years (2), 4–5 years (3), 6–10 years (4) and >10 years in the U.S. (5). “What is your country of origin” was asked with responses corresponding to Iraq (1), Burma (2), and Somalia (3). “What is your religion?” was asked to determine religion. Given the large number of Muslims in the sample, the variable was dichotomized to non-Muslim religion (0) and Muslim religion (1).

General SDoH was assessed by asking about age, sex, socioeconomic status, marital status, and number of years of education. Sex is captured as a dummy variable coded 1 for male and 0 for female. Annual household income is measured as a categorical variable with the following categories: less than $20,000 (1), $20,000–$29,999 (2), $30,000–$39,999 (3), $40,000–49,999 (4), and greater than $50,000 (5). We assessed marital status by asking “Are you married/cohabiting, not married/single, divorced, or widowed?” Responses were dichotomized and coded 1 if the respondent is currently married or living with a partner and 0 otherwise. We operationalized educational attainment with variables coded: 0 years of education (0), 1–5 years of education to reflect grammar school (1), 6–8 years of education to reflect middle school (2), 9–12 years of education to reflect high school (3), 13–16 years of education to reflect a bachelors or professional degree (4) and 17+ years of education to reflect a graduate degree (5).

Neighborhood Characteristics were assessed by asking about engagement with the healthcare system, safety, access to healthy food and access to parks and trails. Engagement with the healthcare system was assessed by asking “Do you have one person you think of as your personal doctor or health care provider?” If “No,” ask: “is there more than one or is there no person you think of as your personal doctor or healthcare provider?” [37] and coded as do not have healthcare providers (0), and have one or more than one healthcare provider (1). Do not know/unsure was coded as missing data. Safety was assessed by asking “Do you have access to safe public exercise or recreational facilities such as walking or running tracks, basketball or tennis courts, swimming pools, sports fields, etc., in your neighborhood?” and coded as no (0) and yes (1). “Don’t know” was coded as missing (3). Access to healthy food was operationalized by asking “How available are affordable fresh fruits, vegetables and other health foods in your neighborhood” [37] and variables were coded not available (0) and available (1). Access to parks and trails was operationalized by asking “Are there any parks or trails in your neighborhood where you can walk, run, or bike?” [37] responses coded as no (0) and yes (1).

#### 2.3.4. Quantitative Data Analysis

Prior to analysis, survey data were exported into SPSS v26 [38] from Qualtrics [30]. Descriptive statistics were calculated to summarize the sample characteristics and distributions of key variables. Continuous variables were assessed for normality and missing data were evaluated for randomness and completeness. Analysis of missing data revealed 4.3% of selected variables from the data set were missing completely at random (Little’s MCAR chi-square test = 286.271, *p* = 0.188). However, extant missing data would result in substantial listwise deletion of cases and diminished statistical power; multiple imputation was used in the reported model. The final hierarchical regression model produced an R^2^ of 0.758, a level of explained variance consistent with the inclusion of multiple sociodemographic and migration-related predictors that are empirically linked to health outcomes. All statistical assumptions, including normality, linearity, homoscedasticity, multicollinearity, and extreme cases, were checked and no problems were found [39]. Estimates were also consistent across all 25 imputed datasets, which supports the stability of the model. Model estimates were consistent across the imputed datasets, further supporting the stability of the results. The researcher used the multiple imputation procedure in SPSS v26 [38] statistical software to impute the missing data. A descriptive analysis of the non-imputed data was first performed to better understand the characteristics of the study sample. To understand the relationship between refugee-specific SDoH, a hierarchical linear regression model was performed with the imputed data using self-reported health status as the dependent variable. All ordinal categorical variables with more than five categories were interpreted as an ordinal approximation of a continuous variable [40]. There were 25 imputed datasets that were created to reduce sampling variability from the imputation process [41]. Imputed independent variables in the model include: marital status, education level, income, employment, social adjustment, years spent in a refugee camp, time spent in the U.S., country of origin, and religion. Access to healthcare, availability of healthy food, access to parks and trails, and safety together were used as predictors but not imputed in the final model. The primary outcome variable—self-reported health status—was treated as a continuous variable (range: 7–39) derived from the validated PROMIS global health scale. Hierarchical linear regression was conducted to examine associations between SDoH and self-reported health. The model was built in three sequential blocks reflecting the conceptual framework: Block 1 included refugee-specific social determinants of health (years in refugee camp, years. in U.S., social adjustment, country of origin, and religion); Block 2 added the general social determinants of health (age, sex, marital status, education, and income); and Block 3, the final model, added neighborhood characteristics (access to: healthcare, healthy food, parks and trails, and safety). Variables were retained in the model based on theoretical relevance and statistical significance (*p* < 0.05). Standardized beta coefficients and confidence intervals were reported for all predictors.

### 2.4. Qualitative Methods

#### 2.4.1. Interview Design and Procedures

In-depth interviews were chosen as the methodology to obtain a textured perspective [42] of health from the Denver refugee community. This qualitative portion of this study was approved by the Colorado Multiple Institutional Review Board (COMIRB Protocol #: 17-0456). The interview guide was developed in collaboration with members of the Community-Based Research Network (CBRN), ensuring cultural appropriateness and relevance. The guide included open-ended questions about participants’ definitions of health, experiences accessing healthcare, and the role of social and community factors in shaping their health.

Interviews were conducted in English, Somali, Burmese, or Arabic by trained bilingual researchers or interpreters trusted by the community. No Karen-speaking participants were interviewed due to the limited availability of a Karen-language interpreter during the qualitative data collection period. Verbal informed consent was obtained from all participants prior to participation. For non-English interviews, a trained interpreter facilitated real-time translation between the English-speaking researcher and participant. With permission, interviews were audio recorded, and the interpreted English was transcribed verbatim by an English-speaking transcriptionist. Interviews lasted approximately 30–60 min, were audio recorded with permission, and transcribed verbatim. All identifying information was removed prior to transcription and participant confidentiality was protected. Participants were compensated $50 USD for their time and contributions.

#### 2.4.2. Interview Sampling and Participants

Participants were purposively sampled to capture variation in country of origin, age, and length of time in the United States. Snowball sampling has been identified as an appropriate sampling method in refugee communities because refugees can be difficult to locate using other means [43]. A total of 27 interviews were conducted, representing roughly equal distribution across the three countries of origin (Iraq, Somalia, and Burma). Recruitment continued until thematic saturation was reached, defined as the point at which no new themes emerged during analysis.

#### 2.4.3. Qualitative Data Analysis

Three trained researchers (two doctoral candidates and one PhD) conducted the qualitative analysis using a constant comparative approach [44,45]. This method involved iterative coding and comparison to identify patterns and relationships across interviews. Analysis proceeded through open coding (initial categorization), axial coding (grouping related codes), and selective coding (identifying core themes).

The team developed an initial codebook based on a priori concepts, supplemented by emergent codes identified during transcript review. Full sentences and paragraphs were used as coding units. Two interviews were double-coded to ensure consistency, and discrepancies were resolved with input from a third researcher [46].

Once the codebook was finalized, all transcripts were coded, and themes were refined. Special attention was given to variations by country of origin, which emerged as a significant predictor of self-reported health in the quantitative analysis. The research team maintained an audit trail of coding decisions and analytic memos throughout the process. Atlas.ti version 8.7.1 [47] was used to manage and organize all qualitative data.

### 2.5. Integration of Methods

Qualitative and quantitative data were collected and analyzed concurrently but independently (Figure 2). After separate analyses were completed, the two strands were integrated using a triangulation/convergence approach in which qualitative themes and quantitative results were compared to assess areas of alignment and divergence [16]. In this design, findings are compared and merged to examine how qualitative themes and quantitative results align or diverge [48]. Integration occurred after the statistical analysis of the survey data and after the qualitative analysis of the text.

Integration involved: (1) identifying key qualitative themes (e.g., how refugees described health); (2) examining significant quantitative results (e.g., associations between country of origin and self-reported health status); and (3) determining points of convergence or contradiction between the two data types. In this design, the quantitative findings provided the strength and direction of associations, while the qualitative findings offered contextual depth and explanation. To support mixed-methods integration, we developed a summary table identifying areas where quantitative and qualitative findings converged, diverged, or offered complementary insights. This table is included in the Results section.

## 3. Results

### 3.1. Quantitative Results

Table 1, Table 2 and Table 3 present the descriptive characteristics of the sample. Participants reported self-reported health status scores ranging from 7 to 30 (M = 24.43, SD = 8.33).

Approximately half of the participants had spent over six years in a refugee camp, and 48% had resided in the United States for fewer than five years. The majority (85%) reported annual household incomes below $30,000, and 70% identified as Muslim.

A hierarchical linear regression was conducted to test the hypothesis that self-reported health status varies based on SDoH, including country of origin. Table 4 shows the results of a hierarchical linear regression analysis for self-reported health status. Refugee-specific SDoH explained 53% of the variance in the model (*p* < 0.001). The addition of general SDoH in Block 2 increased the explained variance by 20.8% (*p* < 0.001). The change in variance explained by adding neighborhood characteristics in the final block was not statistically significant (*p* = 0.169); however, these constructs remain theoretically important and were therefore retained in the final model.

Country of origin remained significantly associated with self-reported health status, with participants from Burma (−3.419, *p* < 0.05) and Somalia (−9.155, *p* < 0.001) reporting significantly lower scores in self-reported health status compared to participants from Iraq. Compared to females, males had a lower self-reported health status (−3.525, *p* < 0.001), controlling for all other independent variables in the model. An increase in age group was significantly associated with higher self-reported health status (1.901, *p* < 0.001) and an increase in education level was significantly associated with a slight decrease in self-reported health status (−0.999, *p* < 0.001), controlling for all other variables. No neighborhood characteristics were significant.

### 3.2. Qualitative Findings

Table 5 presents the characteristics of the interview sample. The refugees interviewed had a mean age of 39 years and had lived in the United States for an average of five years. The majority were female and originally from Iraq. Two overarching themes to describe perceived health emerged consistently across the sample, regardless of country of origin, age, sex, or education level: (1) ability to live life; and (2) happiness. These themes aligned with established theoretical frameworks. Participants’ descriptions of happiness and engagement in daily routines reflect components of the WHO model of well-being, which conceptualizes health as encompassing physical, mental, and social domains [14]. Similarly, the emphasis on safety and community belonging resonates with ecological models of health, which situate individual health experiences within broader environmental and social contexts [3,25,49]. These theoretical connections underscore the multidimensional ways refugees in this study defined and experienced health.

*Theme 1: Ability to Live Life* emerged as all respondents spoke of health as the ability to function daily and participate in everyday activities, from daily responsibilities (childrearing, cooking, cleaning) to recreational activities (going to the park, fishing, religious activities).

Refugees from Burma associated health with the ability to live life through healthy behaviors such as getting enough sleep and exercise and eating well; and carrying out responsibilities such as caregiving, laundry, and work; and participating in leisure activities such as gardening, social and religious events, and going to parks and mountains either alone or with friends and family. It was clear that religion, whether it was Christianity or Buddhism, played a role in health for interviewees from Burma.

*“For me… you have to [be] healthy both spiritual and physical. Sometimes I experience, I have physically, we healthy, we strong enough, but inside, sometimes, we’re not strong…physically we strong, but we are weak in spiritual.”*

*—Respondent from Burma*

Refugees from Somalia associated health with the ability to be an active participant in society. They emphasized the ability to live life through participation in community events, engagement in healthy behaviors, and carrying out daily responsibilities. Notably, the community and familial unit were more prevalent as contributors to perceptions of health with Somali interviewees.

*“…health is people who comes together and greet each other and hug each other, and talk to each other.”*

*—Respondent from Somalia*

*“Anytime I don’t feel sick, I feel happy. If I feel worried about people in Africa, that we left them behind. When you feel something is wrong with your family who is with you right now, that’s when you feel sick”*

*—Respondent from Somalia*

Respondents from Somalia explicitly identified the community as more willing to go to their local community for medical advice than to seek professional medical advice:

*“There’s a lot of people who don’t like to see the doctor unless they are very sick, like an emergency. They just wanna go around and socialize with the community…Just asking questions each other and saying, “How do you stay healthy?” *

*—Respondent from Somalia*

When soliciting perceptions of health from the Iraqi community, the link between mental and physical health was apparent. Healthy behaviors such as exercising, eating well and being able to carry out daily tasks were emphasized. One respondent described their context in Iraq and eloquently expanded upon the direct link between safety, mental health, and physical health:

*“In Iraq, it was everyday story with all the politics and the news and the bombs and the challenges that I had in my life and the threats. It [physical condition] was severe. Now, I think it’s more controllable. My mental health affected my physical health”*

*—Respondent from Iraq*

Safety was also identified as a contributor to health among Somali participants, though it was mentioned less frequently than among Iraqi participants. Notably, Iraqi participants had spent less time in the United States at the time of the interviews compared to participants from Somalia and Burma, which may explain why physical safety emerged as a more immediate concern. Across all three communities, the ability to engage in daily responsibilities and feel a sense of safety were consistently described as components of perceived health.

*Theme II: Happiness* emerged as being highly correlated with health and well-being in the Denver refugee community. Most refugees from Burma associated health directly with happiness. When asking members from the community to describe their health, it was common to receive an initial response such as:

*“When we feel healthy…we are happy.”*

*—Respondent from Burma*

Further probing from this respondent linked the happiness to that made them feel healthy, to the first theme indicated, the ability to carry out daily responsibilities well. Refugees from Somalia also associated health with happiness, which was also repeatedly connected with an absence of worry.

*“I was calm this morning. I didn’t have anything to worry. Yeah, I was just happy this morning. Nothing that I feel like—there’s nothing I’m thinking about.”*

*—Respondent from Somalia*

An emphasis on community and health and happiness emerged consistently across respondents from Somalia.

*They’re happy. The group is happy. You go to the bus and you see someone, and say, “Hi.” They say, “Hi.” *

*—Respondent from Somalia*

Respondents from Iraq also equated health with feelings around happiness:

*Sometimes, when she hear[s] good news, that time she feels happy, when she sleeps good, and she’s not stressed out. There’s nothing that she can think of, and she feels good. That’s when she feels healthy.*

*—Respondent from Iraq*

Happiness was noted as a pervasive theme across countries of origin as a consistent contributor to perceived health.

### 3.3. Qualitative and Quantitative Data Convergence

To further illustrate the integration of findings, Table 6 summarizes areas of convergence across quantitative predictors of self-rated health and qualitative themes emerging from participant interviews.

**Figure 3:**
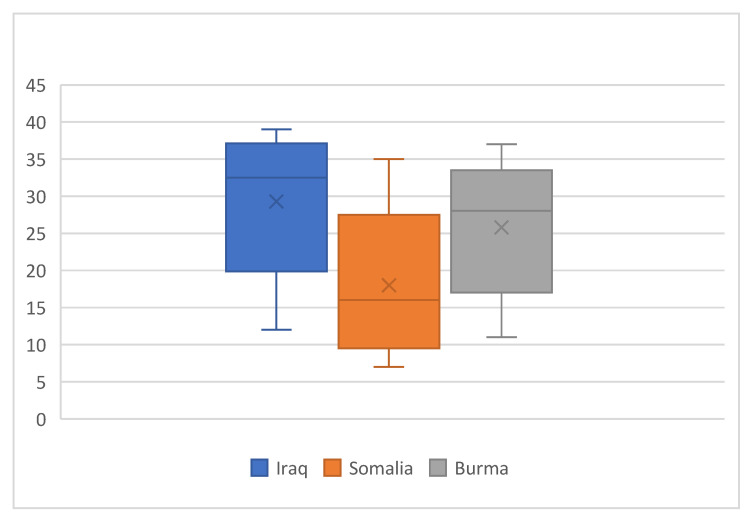
Self reported health status sum score.by country of origin.

In addition to these themes, country of origin emerged as a statistically significant predictor of self-reported health status in the quantitative analysis and was therefore used as an analytic lens for deeper exploration of the qualitative data. Figure 3 illustrates differences in mean self-reported health scores by country of origin and presents these scores alongside community member perspectives. The scale ranges from 7 (lowest self-reported health) to 39 (highest self-reported health). Boxplots display the median (horizontal line) and interquartile range (box); whiskers indicate the minimum and maximum values. Colors denote participants’ country of origin, and the “×” represents the mean. Responses are colored by country of origin as boxplots.

#### 3.3.1. Burma

Refugees from Burma reported a higher mean self-reported health status sum score (26.2, range: 11–37) than those in the sample from Somalia, but lower than those in the sample from Iraq. Exploration of the qualitative data revealed that happiness, the link between physical and mental health, and the ability to carry out daily roles and responsibilities were contributors to overall health and well-being.

*“it’s not just physical health, it will be both physical and mental… I always share my recent trip, that’s why I feel like I wasn’t tired physically or emotionally, and that’s when I feel like that’s what health mean to me, just quiet and enjoy the moment.”*

*—Respondent from Burma*

#### 3.3.2. Somalia

Somali refugees reported the lowest mean health score in the sample (16.5; range: 7–35). A strong sense of community connection emerged as a vital contributor to health and well-being.

*“If people don’t have a relationship, that would be unhealthy. Like if the community don’t have a relationship, they don’t talk to each other. No matter who they are, or where they’re from. The community who lives each other should be talking to each other. They should be knowing each other”*

*—Respondent from Somalia*

#### 3.3.3. Iraq

Iraqi participants reported the highest mean health score (31.2; range: 12–39). Themes included the interdependence of physical and mental health and the importance of safety in shaping a sense of well-being.

*“when [I] hear health, it’s somebody stays in a safe place with good condition, and that’s the important thing.” *

*—Respondent from Iraq*

These qualitative findings provide critical context to the quantitative results and highlight the complex, culturally rooted ways in which refugees conceptualize and experience health.

## 4. Discussion

This study used a community-engaged, mixed-methods approach to explore how resettled refugees understand and describe their own health. The exploratory framework offered in this study is an initial attempt to derive a comprehensive understanding of refugee health. The qualitative findings revealed that constructs such as happiness [3,50] and safety were central to participants’ definitions of health—dimensions that are often absent from standardized health assessments but are increasingly recognized in contemporary research on refugee subjective well-being [51,52]. Studies across diverse displaced populations demonstrate that subjective well-being is shaped by social connection, meaning-making, and psychosocial stability and can mediate relationships between quality of life, safety, and life satisfaction [51,52]. These broader patterns align closely with the themes identified in this study. At the same time, emerging research highlights that refugees’ health perceptions are shaped not only by place-based community relationships but also by digital networks and transnational modes of communication. Social media and digital connectedness have been shown to complement traditional community structures by fostering resilience, sustaining social ties, and supporting health-related knowledge exchange among displaced populations [53]. The findings of this study are situated within a wider landscape in which refugee health is understood as relational, contextual, and increasingly mediated through both in-person and digital forms of connection.

### 4.1. Interpretation of Quantitative Results

Several quantitative findings also merit close attention. Notably, higher educational attainment was associated with *lower* self-reported health. Each increase in education level predicted nearly a one-point decline in perceived health status (−0.999, *p* < 0.001). This counterintuitive result aligns with prior work showing that refugees with higher pre-migration socioeconomic status may experience greater dissonance and acculturative stress upon resettlement [11,54]. This pattern may reflect a mismatch between expectations and post-migration realities, reinforcing the importance of considering social comparison and loss of status in refugee health assessments.

Male participants reported significantly lower self-rated health than females, consistent with global health trends [55]. However, research has suggested that gender differences in self-rated health are complex, with men tending to emphasize physical functioning and women more often highlighting the absence of illness [56]. Other studies have found no gender difference [57], suggesting these patterns may be context-specific and influenced by culturally mediated expressions of distress and well-being.

While aging is typically associated with declining health due to comorbidities [58], older participants reported *higher* self-rated health scores (1.901, *p* < 0.001). This finding may reflect psychological resilience or positive reframing of adversity among older adults [59,60]. It also underscores how self-rated health functions as a culturally embedded perception and construct.

### 4.2. Interpretation of Qualitative Findings and Emergent Constructs of Health

Several constructs—most notably happiness and safety—emerged through the qualitative data but were not included in the initial quantitative survey design.

The centrality of happiness to health perceptions is consistent with a growing body of evidence indicating a bidirectional relationship between health and subjective well-being [61,62]. Despite its prominence in global literature, happiness remains underexplored in refugee health research and rarely appears in standardized survey instruments.

Safety, another salient theme, was not defined merely by the absence of violence but by a holistic sense of security rooted in community, stability, and lived experience. Participants’ expressions of safety often reflected prior exposure to violence, loss, and displacement or contextual dimensions that standard survey tools may not fully capture.

### 4.3. Integration of Mixed-Methods Findings

The integration of qualitative and quantitative approaches expanded the understanding of refugee health and well-being. The three countries of origin represented in this sample reflect diverse pre-migration and migration experiences, including exposure to violence, prolonged displacement, and socioeconomic changes that influence resettlement [63]. This study attempted to capture premigration/migration variation through survey items related to time in refugee camps and change in socioeconomic status. However, country of origin emerged as a strong predictor of self-reported health in both analysis strands, suggesting that both country-level sociopolitical and cultural histories may shape health perceptions.

#### 4.3.1. Burma

Despite the ethnic diversity found in Burma, shared characteristics across cultural groups include an emphasis on family and community and a respect for elders. Burma proved to be an interesting country of origin to define health in this study as the cultural diversity within the country of origin is incredibly rich and ethnicity did not emerge in the qualitative data, nor was it on the survey. However, the qualitative data suggest that health is linked to the ability to carry out daily responsibilities and there is an emphasis on religion (Christianity and Buddhism) as a contributor to perceived health for refugees from Burma living in the Denver metro area.

#### 4.3.2. Somalia

Somali culture is more focused on the family than the individual and it is not uncommon for extended families to live together [64]. The focus on interpersonal relationships and community observed in this study could be leveraged to potentially improve health in the refugee community from Somalia. Nearly all Somalis are Muslim, with less than 1% of the population belonging to other religions [64]. Religion did not emerge in this study as a factor that impacts health and wellbeing. A settings-based public health approach. Ref. [65] suggests that culturally relevant community locations, including mosques, may serve as promising sites for public health programming and interventions. The protracted refugee situation [66] in Somalia is a result of political unrest since the 1990s and, more recently, extreme weather events that have led to internal displacement and food insecurity [67]. It is this context that may contribute to the lower self-reported health status for this country of origin.

#### 4.3.3. Iraq

More than four million Iraqis have been displaced by the war in Iraq [68]. In the aftermath of war, the projected outlook for Iraq is uncertain and there exists the continued need for resettlement assistance for Iraqi refugees [69]. Iraqis had a stable government-funded health care system before the 2003 war and a higher standard of living compared to other refugee groups who lived in refugee camps [11]. This may contribute to the significantly higher self-reported health status in the Iraqi community compared to refugees from Somalia and from Burma. Expectations surrounding education and employment vary widely within refugee communities [70], which may influence adaptation to the resettlement country and well-being.

### 4.4. Implications of Integrated Findings

Engaging the refugee community in this study provided critical insight into how social, mental, and physical health shape overall well-being. Taken together, qualitative and quantitative findings suggest that interventions should be grounded in community-defined perceptions of health. Although experiences varied across countries of origin, specific subgroups demonstrated distinct patterns with practical implications. For example, younger Somali men reported significantly lower self-rated health in the quantitative data, while qualitative findings underscored the importance of community belonging among Somali participants. Together, these patterns indicate that culturally grounded, group-based programs that strengthen social connection may be a promising strategy for supporting this population.

More broadly, the integrated findings point to several directions for NGOs, policymakers, and public health practitioners. Community-grounded approaches are likely to be especially effective when tailored to the cultural, social, and migration histories of specific refugee groups. Programs designed for individuals with higher educational backgrounds should also consider the psychosocial effects of downward mobility and unmet expectations during resettlement. Finally, because participants described safety as stability, predictability, and freedom from chronic stress, interventions should incorporate trauma-informed and contextually grounded strategies that address both emotional and environmental dimensions of well-being.

Finally, this study contributes theoretically by expanding understandings of refugee health beyond clinical and socioeconomic indicators. The findings demonstrate that refugees conceptualize health through subjective well-being, daily functioning, relational safety, and community connection, dimensions that are often overlooked in traditional frameworks. By integrating community-engaged qualitative insights with quantitative associations, this study offers a more holistic and ecologically informed perspective that centers lived experience as foundational to refugee health. This reinforces the value of mixed-methods, community-driven approaches for advancing both theory and practice in refugee health research.

### 4.5. Limitations

Translation quality and interpreter variability remained limitations despite extensive community involvement. Surveys were translated and reviewed in Somali, Burmese, Karen, and Arabic, but nuances may have been lost. Interviews were transcribed in English only, possibly omitting important contextual meaning.

Although the study sought to be inclusive, feasibility and methodological constraints limited data collection to the three largest refugee communities. Respondent-driven sampling requires sufficient population size and network connectivity to produce viable recruitment chains, which smaller groups could not sustain. Additionally, translation and interpretation resources were available only for Somali, Burmese/Karen, and Arabic, further constraining inclusion. Consequently, findings may underrepresent smaller or further minoritized refugee groups in the Denver area.

Recruitment methods (respondent-driven and snowball sampling) did not yield a representative sample, and the overrepresentation of women may introduce bias. While cultural subgroups within countries of origin were not accounted for, this study provided valuable insight into the heterogeneity within national identities. Future frameworks for examining refugee health can consider including: (1) happiness as a social determinant; (2) culturally relevant inquiries around safety; and (3) community-defined neighborhood characteristics.

## 5. Conclusions

This study provides a framework for understanding refugee health through a community-engaged, mixed-methods approach. By integrating self-reported survey data with in-depth qualitative interviews, the study surfaces complex, culturally rooted perceptions of health that go beyond conventional indicators. The mixed-methods design revealed the centrality of constructs such as happiness, safety, and community belonging—factors that may be absent from standardized health assessments but are vital to understanding the lived experiences of refugees.

From a public health perspective, this study emphasizes the need for contextually grounded, community-informed strategies to improve health outcomes among refugee populations. Engaging the refugee community in the design and interpretation of this research not only ensured cultural relevance but also amplified community-defined priorities. This collaborative approach strengthens the foundation for interventions that are not only more effective but also more sustainable and equitable. Institutions seeking to improve refugee health should consider leveraging existing community assets (e.g., religious institutions, peer networks).

Ultimately, this study underscores the value of mixed-methods research for capturing the depth and diversity of refugee health experiences. It offers a model for future studies aiming to reduce health disparities and promotes the co-development of solutions that align with the community’s perceived needs and ultimately improve health outcomes as they are defined by the community.

Future research should build on this work through comparative studies across resettlement sites, refugee groups, and policy environments to assess whether these patterns persist in different sociocultural and geographic contexts. Cross-site replication will help distinguish culturally specific constructs from broader, cross-group dimensions of refugee well-being. Such comparative work can refine theoretical models and support the development of adaptable, equity-focused public health and integration strategies.

## Figures and Tables

**Table 1:** Descriptive Characteristics of Survey Participants: Self-Reported Health Score and Refugee-Specific SDoH.

Demographic Characteristics of Survey Participants				
Variable	Denver-Metro Area Refugee Survey Population (N= 149)			
	Mean	Standard Deviation	Min	Max
**Self-Reported Health Status Sum Score**	24.43	8.33	7	39
**Country of Origin** (n = 149)	**Denver-Metro Area Refugee Survey Population (n = 149)** Frequency (%)			
Burma	51 (34.2)			
Iraq	46 (30.9)			
Somalia	52 (34.9)			
**Years in Refugee Camp** (n = 129)				
0	32 (24.8)			
1 to 5	32 (24.8)			
6 to 10	29 (22.5)			
11 to 15	19 (14.7)			
16+	17 (13.2)			
**Time in the United States** (n = 146)				
<1–3 years	33 (22.6)			
4–5 years	37 (25.3)			
6–10 years	51 (34.9)			
11+ years	25 (17.1)			
**Social Adjustment** (n = 129)				
No change	88 (68.2)			
Positive change	22 (17.1)			
Negative change	19 (14.7)			

**Table 2:** Descriptive Characteristics of Survey Participant: General SDoH.

Demographic Characteristics of Survey Participants	
Variable	Denver-Metro Area Refugee Survey Population (N = 149)
**Sex** (n = 135)	
Male	44 (32.6)
Female	91 (67.4)
**Age** (n = 145)	
18–25	29 (20.0)
26–39	51 (35.2)
40–54	26 (17.9)
55–64	19 (13.1)
65+	20 (13.8)
**Employment Status** (n = 141)	
Employed for wages or self-employed	63 (44.7)
Not Employed	78 (55.3)
**Marital Status** (n = 134)	
Married/Cohabiting	81 (60.4)
Single/Divorced/Widowed	53 (39.6)
**Education** (n = 130)	
None	21 (16.2)
Elementary School	16 (12.3)
Middle School	13 (10.0)
High School	44 (33.8)
Bachelor’s Degree	30 (23.1)
Advanced Degree	6 (4.6)
**Household Income** (n = 141)	
<$20,000	85 (60.3)
$20,000–$29,999	35 (24.8)
$30,000–$39,999	7 (5.0)
$40,000–$49,000	6 (4.3)
>$50,000	8 (5.7)

**Table 3:** Descriptive Characteristics of Survey Participant: Neighborhood Characteristics.

Demographic Characteristics of Survey Participants	
Variable	Denver-Metro Area Refugee Survey Population (N = 149)
**Access to a Healthcare Provider** (n = 144)	
No	29 (20.1)
Yes, one healthcare provider	69 (47.9)
Yes, more than one	24 (16.7)
Don’t know/not sure	22 (15.3)
**Access to Parks and Trails** (n = 145)	
Yes	114 (78.6)
No	31 (21.4)
**Religion** (n = 149)	
Muslim	104 (69.8)
Other religions (e.g., Christian)	45 (30.2)
**Access to Healthy Food** (n = 147)	
Yes	136 (92.5)
No	11 (7.5)
**Safe Public Spaces (Safety)** (n = 129)	
Yes	94 (72.8)
No	35 (27.1)

**Table 4:** Summary of Hierarchical Regression Analysis for Variables Predicting Self-Reported Health Status Sum Score in the Denver Metro Area Refugee Community.

N = 135		
Model Fit	R^2^ = 0.758	F = 18.950 (*p* < 0.001)
Independent Variables	B (*p*-Value)	CI
Years spent in a refugee camp	0.485 (0.269)	(−0.380, 1.350)
Length of time in US	−0.604 (0.114)	(−1.354, 0.147)
Social Adjustment (Positive Change)	−0.632 (0.671)	(−3.575, 2.311)
Country of Origin: Burma	−3.419 (0.030) *	(−6.493, −0.345)
Country of Origin: Somalia	−9.155 (<0.001) ***	(−12.258, −6.052)
Religion: Muslim	0.255 (0.813)	(−1.880, 2.390)
Age	1.901 (<0.001) ***	(1.105, 2.698)
Sex: Male	−3.252 (<0.001) ***	(−5.158, −1.345)
Marital Status: Coupled	0.348 (0.692)	(−1.383, 2.079)
Education Level	−0.999 (<0.001) ***	(−1.584, −0.414)
Income	−0.189 (0.630)	(−0.966, 0.587)
Employment	−1.757 (0.075)	(−3.692, 0.178)
Access to Healthcare	0.917 (0.511)	(−1.836, 3.670)
Access to Healthy Food	2.606 (0.106)	(−0.566, 5.778)
Access to Parks & Trails	−0.208 (0.855)	(−2.455, 2.038)
Safe Recreation	−1.915 (0.080)	(−4.067, 0.236)

Note: Reference categories include country of origin: Iraq, Sex: Male, Religion: Muslim, Employment: Employed for wages, Healthcare: Access to healthcare provider * *p* < 0.05; *** *p* < 0.001. Note: Results are based on an imputed dataset. Missing data were handled using multiple imputation with 25 imputations.

**Table 5:** Descriptive Characteristics of Interview Participants.

Variable	Interview Participants (n = 27)	
	Mean	Standard Deviation
**Age**	39.3	14.7
**Years in US**	4.8	4.8
**Sex**	Frequency (%)	
Male	7 (25.9)	
Female	20 (74.1)	
**Country of Origin**	Frequency (%)	
Iraq	11 (42)	
Burma	8 (29)	
Somalia	8 (29)	

**Table 6:** Integration of Quantitative and Qualitative Findings.

Quantitative Finding	Related Qualitative Theme(s)	Convergence	Interpretation
Older age associated with higher self-rated health	Older adults describe stability, acceptance, and a strong sense of resilience; value routines that support well-being	✔	Qualitative accounts of resilience and reframing of adversity align with higher self-reported health among older participants
Men report lower health than women	Men emphasize stress related to employment, financial responsibility, and role expectations	✔	Men’s narratives of stress and pressure mirror lower self-rated health scores in quantitative data
Higher education associated with lower self-rated health	Educated refugees describe downward occupational mobility, unmet expectations, and stress navigating new systems	✔	Qualitative themes illuminate why education is associated with perceived health, despite typically protective effects
Somali and Burmese origin predict lower health (compared to Iraq)	All interviewees describe trauma, resettlement hardship, and barriers related to environment and the health system	✔	Quantitative differences between groups reflect lived experiences described in interviews
No significant quantitative associations with SDoH indicators (e.g., access to parks, food, healthcare)	Participants emphasize happiness, daily routines, safety, community belonging, and emotional well-being as core components of health	—	Qualitative data expand beyond measured SDoH variables, highlighting culturally grounded definitions of health not captured in the quantitative model

*Note:* ✔ indicates convergence between quantitative and qualitative findings identified during the integration phase.

## Data Availability

The datasets generated and analyzed during the current study are not publicly available due to confidentiality agreements with participants and community partners. However, de-identified data supporting the findings of this study may be made available from the corresponding author upon reasonable request.

## Abbreviations

The following abbreviations are used in this manuscript:

SDOH	Social Determinants of Health
